# Myoclonus from Antibiotic Therapy (Ceftazidime-induced Neurotoxicity): A Case Report and Review

**DOI:** 10.7759/cureus.2250

**Published:** 2018-03-01

**Authors:** Chong Yau Ong, Yan Qin

**Affiliations:** 1 Family Medicine, Sengkang General Hospital, Sengkang Health, Singhealth, Singapore; 2 Department of Internal Medicine, Singapore General Hospital

**Keywords:** myoclonus, ataxia, ceftazidime, antibiotics, neurotoxicity

## Abstract

A 78-year-old Chinese man with a history of end-stage renal disease (ESRD) presented with fever of one-day duration. He was treated for catheter-related sepsis with intravenous piperacillin and tazobactam, which was later switched to vancomycin and ceftazidime secondary to persistent fever with negative cultures. On the fifth day of treatment with vancomycin and ceftazidime, he developed new-onset upper limb myoclonus which progressed to bilateral upper limb ataxia. A provisional diagnosis of myoclonus and ataxia secondary to neurotoxicity related to ceftazidime was made and the ceftazidime was ceased. His symptoms resolved over three days and he returned to his baseline neurological status by day 5 following cessation.

## Introduction

Ceftazidime, like other cephalosporins, is a commonly used antibiotic in the hospital setting. However, cephalosporin-induced neurotoxicity is not well recognized, and is likely under-reported especially amongst patients with chronic kidney disease. We present a case of a 78-year-old man who developed myoclonus and ataxia secondary to ceftazidime.

## Case presentation

A 78-year-old Chinese man presented to the emergency department with a fever of one day’s duration. He had a history of end-stage renal disease (ESRD) secondary to hypertensive nephrosclerosis. He was on regular haemodialysis via permanent catheter after the stenosis of his left arteriovenous fistula, and was awaiting fistuloplasty. Comorbidities included dyslipidaemia, diabetes mellitus, antral gastritis and iron deficiency anaemia. He had no localizing symptoms for the fever and there was no contact or travel history. The examination was unremarkable and he was treated for catheter-related sepsis with intravenous piperacillin and tazobactam (2.25 g every 6 hours). However, he had persistent fever for one week, and repeated blood and urine culture yielded no growth of microorganisms. Computed tomography of thorax, abdomen, and pelvis found no source of infection. A transthoracic echocardiogram showed no vegetation. The infectious diseases team was consulted for pyrexia of unknown origin. Antibiotic therapy was switched to vancomycin and ceftazidime (2 g daily with 1 g top up after each dialysis, with the intention to treat presumptive melioidosis; an endemic soil-borne disease in Southeast Asia region). The catheter was removed after fistuloplasty was performed and its tip was sent for culture, which yielded no growth.

On the fifth day of treatment with the changed antibiotics regimen (vancomycin and ceftazidime), he developed new onset of myoclonus of the upper limbs bilaterally. He had no change in mental status suggestive of an encephalopathy. Prior to this, he had no such movement disorder or any neurological symptoms. He then progressed to develop bilateral upper limbs ataxia. There were myoclonic movements of the upper limbs. The myoclonic movements were less prominent in the lower limbs. There was no apraxia or pseudo-athetosis. No truncal ataxia was observed. Mild right proximal hemiparesis (power 4 over 5 [Medical Research Council (MRC) Scale]) was noted when compared with the contralateral limbs.

Magnetic resonance imaging (MRI) of the brain showed no acute intracranial haemorrhage or ischemia. A renal panel was not suggestive of uremic encephalopathy. The ammonia level and liver function tests were within normal limits. A provisional diagnosis of myoclonus and ataxia secondary to neurotoxicity due to the use of ceftazidime on a background of ESRD was made, and the ceftazidime was ceased. The patient’s neurological symptoms resolved over 72 hours and he was at his baseline by five days. Electroencephalography (EEG) was scheduled but not done as his symptoms resolved completely.

Vancomycin was continued and amikacin was added following cessation of ceftazidime. The patient was on series of antibiotics for one month but still had intermittent fever despite all the cultures were negative. Parasite infection was suspected subsequently. Three sets stool ova/cyst/parasite were sent and did not yield any parasites or strongyloides. Ivermectin, a medication that is effective against many types of parasites, were given for two days as an empirical treatment. The patient’s fever subsided after ivermectin.

We searched the literature and noted several publications on neurotoxicity from cephalosporins. Search terms in PubMed were cephalosporin or ceftazidime and neurotoxicity (including myoclonus, seizure, and encephalopathy). The literature search was also extended to the Google search engine and five more case reports obtained. Reports on neurotoxicity from ceftriaxone alone and cefepime alone were not included so that the clinical details were comparable to our patient who received ceftazidime. All case reports that were selected were written in English and involved human subjects. We limited the search to publications from the year 2000. Clinical details such as demographic data, disease pattern, and EEG findings were obtained. Clinical data of 10 patients with ceftazidime-induced neurotoxicity from nine publications were summarised in Table 1.

**Table 1:** Clinical details of patients with ceftazidime-induced neurotoxicity. AED: Antiepileptic; BD: Twice daily; CAPD: Continuous ambulatory peritoneal dialysis; ESRD: End stage renal disease; f: Frequency; g: gram; H: Hertz; HD: Haemodialysis; IV: Intravenous; IP: Intraperitoneal; OM: Once in the morning; NA: Not available; QD: Four times a day; STAT: Immediately; TDS: Thrice daily.

Authors	No. of patients	Age/sex	Creatinine (µmol/L)	Dose	Indication	Clinical features	EEG	Latency (days)	Resolution (days)
Martinez, et al. (2001) [1]	2	64/M	707.2	IV 2 g OM	Pneumonia	Agitation, confusion, myoclonus	Continuous generalised 12 Hz sharp wave	8	2 (improved)
		38/M	592.3	IV 2 g OM	Pneumonia	Confusion, myoclonus	Continuous, generalised 12 Hz sharp wave	5	2 (improved)
Chow, et al. (2003) [2]	1	62/F	901.7	IP 250 mg QD x 11 days then IV 1 g QD x 5days	CAPD peritonitis	No verbal response	Increased theta activity triphasic waves	2	NA
Chuang, et al. (2003) [3]	1	76/F	698.3	IV 2 g BD	Pseudomonas wound	Altered consciousness, myoclonus upper limbs	Short interval diffuse discharge (PSIDD)	5	3
Primavera, et al. (2004) [4]	1	72 /F	424.3	IV 4 g/day	Peritonitis	Mood change, anxiety, mute, extrapyramidal signs, myoclonic jerks	Generalised sharp waves	3	2
Martin (2007) [7]	1	43/M	ESRD	IV 1g daily	Pneumonia	Dysarthria, confusion, no verbal response, facial myoclonic jerks	NA	5	6
Chan, et al. (2006) [5]	1	65/F	NA	IV 2 g BID	Pneumonia	Confusion, asynchronous myoclonus	NA	2	7
Vannaprasaht, et al. (2006) [6]	1	70/F	ESRD	IV 1 g BD then IP 1.5 g/day then IP 11 g/day x2day	Peritonitis	Altered conscious level, mutism, asterixis, nystagmus	Generalised 3 spikes-and-wave	2	6 (with HD and AED)
Joseph, Vimala (2015) [8]	1	49/M	ESRD	IV Ig BD	Malignant otitis media	Myoclonus (generalised), Altered sensorium	NA	2	5
Haldar, et al. (2015) [9]	1	14/M	Normal	IV 1.5 mg STAT	Preoperative induction	Generalised seizures	NA	5	Minutes (with midazolam)

## Discussion

In addition to the patient that we described, neurotoxicity effects of ceftazidime were also reported in 10 patients from nine publications. Among these 10 patients, eight had underlying renal impairment (mostly end-stage renal disease on renal replacement therapy), one patient’s creatinine level was normal, and the remaining one patient’s creatinine level was not available.

The median age of all the patients was 65 years. The indications for treatment requiring ceftazidime among the patients included lower respiratory tract infections/pneumonia (four patients), peritonitis (three patients), pseudomonas wound infection, malignant otitis media, and preoperative induction.

EEG findings of six patients were available [1-3,4,7]. Five of them demonstrated epileptic activity including continuous, generalised sharp waves and periodic short interval diffuse discharge (PSIDD). One showed increased theta activity with triphasic waves [2].

Since ceftazidime is not metabolised in the body and is excreted unchanged in the active form in the urine by glomerular filtration, patients with renal impairment are more vulnerable to the ceftazidime neurotoxicity even if it is given at recommended doses. Intraperitoneal ceftazidime is the drug of choice in peritonitis and this mode of delivery augments the above observation. The increase in permeability of the peritoneal membrane during infection may explain the increased systemic toxicity. The association between neurotoxicity and ceftazidime is not completely understood.

The neurological manifestations from ceftazidime toxicity varied from confusion or hallucinations to marked signs of myoclonus and seizures. Our patient has a progression to ataxia without encephalopathy and this has not been described in published reports. The convulsive activity or epileptogenic effect of cephalosporins involves inhibition of gamma-aminobutyric acid (GABA) binding to GABA (A) receptors. The latency in the development of the neurological symptoms ranged from few minutes to 15 days. The resolution of symptoms typically occurs within two to seven days of discontinuation of ceftazidime. Although outcomes were not available in one case report, it seems that such neurotoxicity is reversible.

## Conclusions

Antibiotic-induced neurotoxicity is often overlooked or misinterpreted despite extensive administration of these agents. Early recognition of this condition and withdrawal of offending antibiotics is therefore of significant clinical importance. It may prevent unnecessarily invasive investigations and serious complications in these already complex renal patients. Patients presenting with confusion, speech disturbances, temporospatial disorientation and other forms of altered mentation should have an EEG assessment, as these antibiotics may precipitate seizures even in patients who do not have a predisposition to seizures or known epilepsy. The diagnosis of non-convulsive status epilepticus (NCSE) may be missed if an EEG is not performed. As the use of ceftazidime and other cephalosporins (especially ceftriaxone and cefepime) is becoming more common, clinicians and physicians ought to have heightened awareness of their potential neurotoxicity in this context.
